# Artesunate plus sulfadoxine-pyrimethamine for treatment of uncomplicated Plasmodium falciparum malaria in Sudan

**DOI:** 10.1186/1475-2875-4-41

**Published:** 2005-09-14

**Authors:** Sakina B Elamin, Elfatih M Malik, Tarig Abdelgadir, Ammar H Khamiss, Mamoun M Mohammed, Elderderi S Ahmed, Ishag Adam

**Affiliations:** 1 NationalMalaria Control, Ministry of Health, Khartoum, Sudan; 2 Albayan College for Science, Sudan University for Science and Technology, Sudan; 3 University of Kassala, Sudan; 4 Faculty of Medicine University of Khartoum, The Academy of Medical Sciences and Technology, Department of Obstetrics & Gynecology, Faculty of Medicine University of Khartoum, P. O. Box 102, Khartoum, Sudan

## Abstract

**Background:**

Early diagnosis and effective treatment with an appropriate drug form the main components of the World Health Organization's strategy to reduce malaria related mortality. The few available drugs might be safeguarded if combined with artesunate. The addition of artesunate to a standard antimalarial treatment substantially reduces treatment failure, recrudescence and gametocyte carriage.

**Methods:**

During late 2004, the efficacy of artesunate (4 mg/kg. day, on days 0–2) plus sulfadoxine-pyrimethamine (25 mg/kg, on day 0) for the treatment of uncomplicated *Plasmodium falciparum *malaria was investigated in four sentinel areas in Sudan, with different malaria transmission (Damazin, Kassala, Kosti, and Malakal).

**Results:**

Two hundreds and sixty-nine patients completed the 28-day follow-up. On day one, 60 (22.3%) patients were febrile and 15 (5.5%) patients were parasitaemic. On day three, all the patients were afebrile and aparasitaemic. While two patients (0.7%, Kassala) showed late Clinical and Parasitological Failures, the rest (99.3%) of the patients demonstrated Adequate Clinical and Parasitological Response. A gametocytaemia were detected during the follow-up in one patient (0.37%, Kassala). Adverse drug effects were detected in 32 (11.9%) patients

**Conclusion:**

The study showed that AS plus SP is an effective, safe drug in the treatment of uncomplicated *P. falciparum *malaria in Sudan.

## Background

There are almost 515 (range 300–660) million episodes of clinical *Plasmodium falciparum *malaria infections [1]. Drug-resistant malaria is spreading in Africa and countries with high levels of resistance have witnessed increased morbidity and mortality [2]. Early diagnosis and effective treatment with an appropriate drug form the main components of the World Health Organization's strategy to reduce malaria-related mortality [3].

The few available drugs might be safeguarded if combined with artesunate. The addition of artesunate to standard antimalarial treatments substantially reduces treatment failure, recrudescence and gametocyte carriage, preventing the emergence and spread of drug resistance and interrupting the transmission of *P. falciparum*. Coupled with early detection and confirmed diagnosis, this strategy represents the only way forward in the chemotherapy of malaria [4-8].

Malaria causes between 7.5 to 10 million cases and 35,000 deaths every year in Sudan [9]. Due to the spread of multidrug-resistant *P. falciparum *malaria in Sudan [10,11], artesunate plus sulfadoxine-pyrimethamine is recommended as the first-line treatment for uncomplicated *P. falciparum *malaria. The study aimed to investigate the efficacy of AS plus SP, as there is little published data in Sudan [8,12].

## Patients and methods

### Data collection

**Figure 1:**
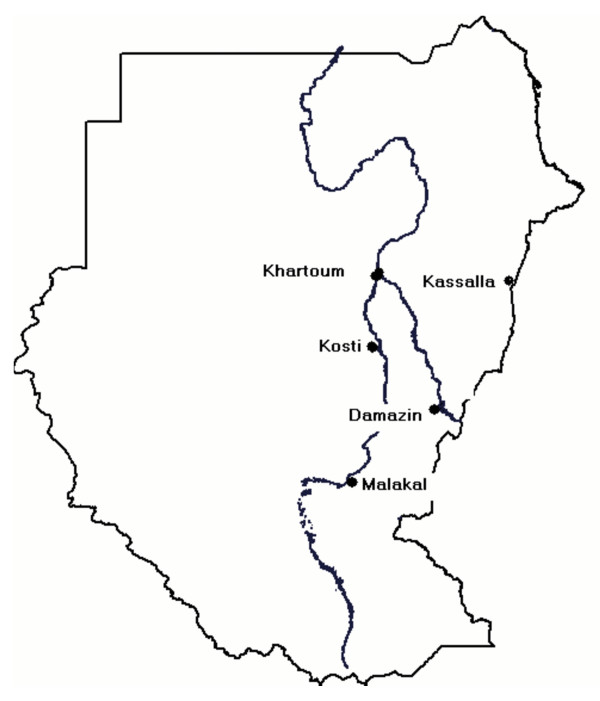
A sketch map of the Sudan, showing the main rivers, Khartoum and the four sites where the study was conducted.

The study was conducted in October and November, 2004 at four health centres in different regions of Sudan (Damazin, Kassala, Kosti, and Malakal) (Figure 1). Three of these areas were characterized by low malaria transmission and the fourth (Malakal) was characterized by stable transmission [13]. Febrile (temperature ≥ 37.5°C) patients with uncomplicated *P. falciparum *malaria [14], who had no history of antimalarial drug use during the preceding two weeks, were recruited for the study. Pregnant women and patients with mixed infections were excluded.

After obtaining informed consent from the patient or the child's parents, a fixed questionnaire including relevant socio-demographic characteristics, medical history, physical findings and investigations conducted was completed for each patient.

### Laboratory methods

Blood films were prepared, stained with Giemsa and 100x oil immersion fields were examined. The parasite density was counted against 200 leucocytes, assuming 6,000 leucocytes/μl. All the slides were double-checked blindly and only considered negative if no parasites were detected in 100 oil immersion fields. If gametocytes were seen, then the count was extended to 500 leucocytes.

## Treatment and follow up

The patients were given the AS plus SP combination, with artesunate (4 mg/kg. day) given on days 0–2 and a single dose of SP (25 mg/kg) (Dafra Pharma, Beerse, Belgium) given on day 0. The tablets were crushed and dissolved in water for children who were not able to swallow them. Subjects were observed for vomiting for one hour; the full dose was repeated for those who vomited within 30 min and half of the dose was repeated if vomiting occurred between 30 and 60 minutes.

### Follow-up and re-treatment

Patients were requested to come on days 1, 2, 3, 7, 14, 21 and 28 and at any time if they felt unwell. At each visit, body temperature was measured and blood films were prepared. During the follow-up the patients were asked if they suffered from side effects which can be expected from antimalarial treatment (nausea, vomiting, abdominal pain, dizziness and rash); these symptoms were considered to be drug related if they had not been reported at the patient's first presentation in the clinic.

Quinine was given for treatment failures. Early Treatment Failures (ETF) in case of significant parasitaemia at day 2 or 3 or parasites and fever at day 3. Late Clinical Failures (LCF) for cases with parasites and fever during follow-up after day 3 and Late Parasitological Failures (LPF) for parasite infections with/without fever during the follow-up. Cases which remained negative during follow-up were considered Adequate Clinical and Parasitological Responses (ACPR). These were modified WHO guidelines [14,15].

### Statistics

Data were entered into a computer database and SPSS software (SPSS Inc., Chicago, IL, USA) was used for statistical analysis. The means (age, weight, temperature and parasite count) were calculated for all the patients and were compared between the patients in the different locations using one way analysis of the variance (ANOVA), when the data is normally distributed and by the Kruskal Wallis test if the data was not normally distributed. Percentages were calculated and compared for the patients in the four locations by an χ^2 ^test. *P *< 0.05 was regarded significant.

### Ethical clearance

The study received ethical clearance from the Sudanese National Malaria Administration.

## Results

Two hundred and ninety (32.5%) out of 890 screened patients fulfilled the criteria and were enrolled in the study. Twenty-one (7.2%) of these were lost in the follow-up and 269 patients (72, 50, 70, and 77 from Damazin, Kassala, Kosti, and Malakal, respectively) completed the 28-day follow-up. Their different characteristics are shown in Table 1. The mean age and weight were significantly higher in the Kassala group. The parasite count was significantly higher in the Malakal area. 37.0% (100 patients) were children less than five years old; this proportion was significantly higher in Malakal group (92.2%, see Table 1). One hundred and twenty seven subjects (47.2%) were females; their percentages were not significantly different within the groups (see Table 1).

**Table 1:** The base line (day 0) characteristics of the 269 patients who completed the 28 days of follow-up after the treatment with artesunate plus sulfadoxine-pyrimethamine*.

Variable	Total (N = 269)	Damazin (*N = 72*)	Kassala (*N = 50*)	Kosti (*N = 70*)	Malakal (*N = 77*)	Significance
Age, years	12.1 (12.4)	10.6 (9.0)	24.3 (15.4)	14.7 (12.08)	3.4 (1.2)	P < 0.05
Weight, Kg	28.1 (19.3)	25.6 (14.5)	47.4 (21.5)	33.2 (18.5)	3.2 (0.36)	P < 0.05
Temperature, °C	38.2 (0.76)	38.1 (0.6)	38.2 (0.6)	37.9 (0.5)	38.6 (0.9)	P > 0.05
Parasite count, rings/μ	25532.3 (24196.2)	21924.3 (17748.7)	32240.3 (28372.9)	11509.8 (11135.2)	37297.5 (27844.5)	P < 0.05
Children < 5 years	100 (37.0)	12 (16.7)	5 (10)	12 (17.1)	71 (92.2)	P < 0.05
Female	127 (47.2)	37 (51.4)	18 (36)	32 (45.7)	40 (51.9)	P > 0.05

*Data were shown as mean (SD) or numbers (%) as appropriate

On day one, 60 (22.3%) patients were febrile and 15 (5.5%) patients were parasitaemic. By day three all the patients were afebrile and aparasitaemic. There were two (0.7%) Late Clinical and Parasitological Failures (days 7 and 22) from Kassala, there was no Clinical and Parasitological Failures from other locations (Table 2). Only one patient (Kassala) showed gametocytaemia on day 14 of the follow-up. Thirty two (11.9%) patients suffered expected adverse effects (nausea, itching and dizziness), but these were mild and resolved spontaneously.

**Table 2:** Trail profile, showing number of patients enrolled, treated and completing the 28 days of follow-up after the treatment with artesunate plus sulfadoxine-pyrimethamine*.

Variable	Total	Damazin	Kassala	Kosti	Malakal
The recruited patients	290	77	53	76	84
Lost to follow-up	21 (7.2)	5 (6.5)	3 (5.6)	6 (7.9)	7 (8.3)
					
Patients completed the 28-days of follow-up	269 (94.8)	72 (93.5)	50 (94.4)	70 (92.1)	77 (91.7)
Accurate clinical and parasitological response	267 (99.3)	72 (100)	48 (96)	70 (100)	77 (100)
Late clinical and parasitological failure	2 (0.7)	0 (0)	2 (4)	0 (0)	0 (0)

*Data were shown as numbers (%).

## Discussion

The study investigated the efficacy of AS plus SP for the treatment of uncomplicated *P. falciparum *malaria at four sites in the Sudan. This is probably the largest study reporting AS plus SP efficacy in Sudan until now. Although, the baseline characteristics (age and parasite count) were significantly different between the four locations, the study showed that two (0.7%, Kassala) out of 269 patients were found to have Late Clinical and Parasitological Failures. Since the parasite genotyping (PCR) was not conducted, the possibility of re-infection/recrudescence is still there. Hundred percent efficacy of AS plus SP was recently reported from eastern Sudan [8] and 99% from southern Sudan [12]. The high cure rate in this study is comparable to that reported from neighbouring African countries [5,6]. However, the highest drug resistant *P. falciparum *strains were reported from eastern Sudan [11,16]. The expected adverse effects (nausea, itching and dizziness) were reported in (11.9%) of the patients in this study. These results were in line with, observations of others, where the adverse effects were not significantly different, if compared with those of SP alone [5,8].

The adverse effects (nausea, vomiting) might influence the adherence to AS plus SP, especially science this therapy is only available in the oral form, which is not the medication preferred by Sudanese patients [17]. Furthermore, adherence may be influenced by the multiple doses of the combination, rather the previous single dose of SP, which was reported to be the most important single factor for the best adherence of SP among Sudanese patients [17].

A post- treatment gametocytaemia was detected in one patient in Kassala area. High (20%) levels of gametocytaemia had been reported in the eastern Sudan following SP, quinine and mefloquine treatment [18-20]. However, it has not been reported during the follow-up of patients in the eastern Sudan treated with artemether, artesunate plus mefloquine or AS plus SP [8,19,21]. The ability of artesunate to reduce the post-treatment gametocytaemia is important, as it may reduce transmission [4].

## Conclusion

The study showed that, As plus SP is an effective, safe drug in the treatment of uncomplicated *P. falciparum *malaria in Sudan.

## Authors' contributions

SBE, EMM, TA, MTM, ESA carried out the study in the different sites and participated in the statistical analysis and procedures, AHK participated in the statistical analysis, IA coordinated and participated in the design of the study, statistical analysis and the drafting of the manuscript. All the authors read and approved the final version.
